# Regio- and stereoselective oxidation of unactivated C–H bonds with *Rhodococcus rhodochrous*

**DOI:** 10.3762/bjoc.8.56

**Published:** 2012-04-03

**Authors:** Elaine O’Reilly, Suzanne J Aitken, Gideon Grogan, Paul P Kelly, Nicholas J Turner, Sabine L Flitsch

**Affiliations:** 1 School of Chemistry, Manchester Interdisciplinary Biocentre, The University of Manchester, 131 Princess Street, Manchester, M1 7ND, UK, Fax: +44 (0)161 2751311, Tel: +44 (0)161 3065172; 2 Department of Chemistry, University of York, Heslington, York, YO10 5DD, UK, Tel: +44 (0)1904 328256

**Keywords:** biocatalysis, cytochrome P450, hydroxylation, *Rhodococcus rhodochrous*, selective C–H activation

## Abstract

The ability of *Rhodococcus rhodochrous* (NCIMB 9703) to catalyse the regio- and stereoselective hydroxylation of a range of benzyloxy-substituted heterocycles has been investigated. Incubation of 2-benzyloxytetrahydropyrans with resting cell suspensions of the organism yielded predominantly a mixture of 5-hydroxylated isomers in combined yields of up to 40%. Exposure of the corresponding 2-benzyloxytetrahydrofuran derivatives to the cell suspensions gave predominantly the 4-hydroxylated isomers in yields of up to 26%. Most interestingly, 2-(4-nitrobenzyloxy)tetrahydrofuran and 2-(4-nitrobenzyloxy)tetrahydropyran were transformed in high yields to the 4-hydroxylated and 5-hydroxylated products, respectively.

## Introduction

Selective C–H activation remains a challenge for synthetic chemists, who often rely on differences in the steric and electronic properties of bonds to achieve regioselectivity [1]. The preparative-scale generation of hydroxylated intermediates can often provide synthetically useful derivatives as well as pharmaceutically important drug metabolites and lead compounds [2–6]. Difficulties associated with chemical routes for the stereo- and regioselective preparation of such compounds has led to the exploitation of natural and recombinant whole-cell biocatalysts as tools for their preparation [7–11].

Many hydroxylating enzymes have been identified within bacteria belonging to the genus *Rhodococcus*. These organisms are ubiquitous in the environment and their ability to activate recalcitrant compounds and xenobiotics with an array of mono- and dioxygenases has made them the focus of research into bioremediation and biocatalyst development [12]. The soil bacterium *Rhodococcus rhodochrous* (also referred to as *Corynebacterium* sp. 7E1C, *Rhodococcus rhodochrous* ATCC 19067 and *Gordonia rubripertinctus* 7E1C) is known to oxidise a wide variety of aliphatic hydrocarbons, including *n*-alkanes and isoalkanes, and has been reported to catalyse the terminal hydroxylation of chloroalkanes and long-chain alkenes [13]. It has been suggested that this strain contains an alkane-inducible NADH-dependent hydroxylase that catalyses the oxidation of *n*-octane to 1-octanol [13–14]. In light of this, we have explored the ability of this strain to perform regio- and stereoselective oxidations of selected protected heterocyclic substrates. Protected substrates are of particular interest, as subtle changes in the protecting group have been shown to affect the regioselectivity of hydroxylation [10].

**Scheme 1:**
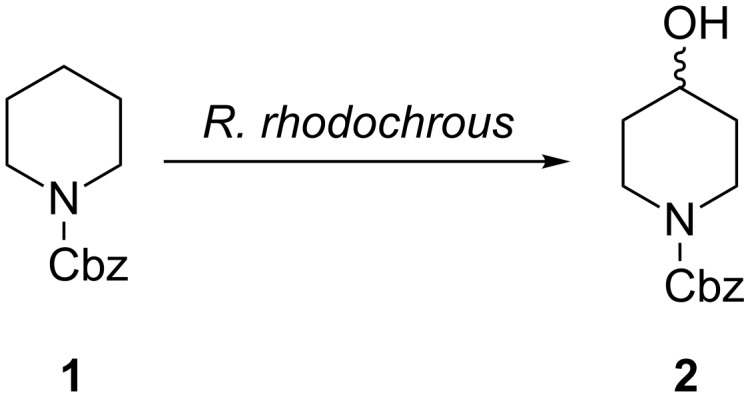
Regioselective hydroxylation of Cbz-piperidine by *Rhodococcus rhodochrous* resting cells.

An initial substrate screen with the organism revealed that carbobenzyloxy-protected (Cbz) piperidine **1**, which is known to be hydroxylated by various microorganisms [15], was oxidised selectively in the 4-position, as indicated in Scheme 1 [10].

This encouraging result prompted us to challenge this organism with a variety of novel heterocyclic substrates. Substrates based on oxygen heterocycles were thought to be particularly interesting since, upon deprotection, they would yield polyfunctionalised molecules that represent valuable chiral intermediates. A number of *p*-substituted benzyloxytetrahydropyrans and furans were synthesised and incubated with resting cell suspensions of *R. rhodochrous*. Our aim in this study was to establish whether the organism is capable of catalysing the hydroxylation of this panel of compounds and to investigate whether these hydroxylations proceeded with regio-, diastereo- and enantioselectivity. We were also interested in establishing whether the oxidising capabilities of this organism were the result of cytochrome P450 activity.

## Results and Discussion

### Transformations of THP ether derivatives

**Scheme 2:**
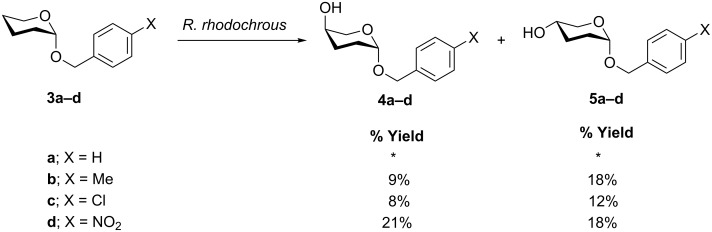
Results from the incubation of THP ether derivatives with *Rhodococcus rhodochrous* showing the isolated yields; *an inseparable mixture of products was obtained.

The results of the biohydroxylations of the *p*-substituted 2-benzyloxypyrans **3a–d** are summarised in Scheme 2.

The regiochemistry and relative stereochemistry of the biohydroxylated products were determined by analysis of ^1^H NMR coupling constants and by COSY and NOE/NOESY experiments, which identified the 5-*axial* and 5-*equatorial* hydroxylated 2-benzopyran isomers **4** and **5** as the major products.

We initially examined the regioselectivity of the biohydroxylation of tetrahydropyran (THP) derivative **3a** and found that an inseparable mixture of hydroxylated products was obtained. Studies have shown that minor alterations in substrate structure can strongly affect the selectivity of *Beauveria bassiana* ATCC 7159 [16–17]. Interestingly, the introduction of substituents on the aryl ring of some substrates can assist in directing hydroxylation away from the substituted position and can improve regioselectivity. For example, varying the aryl ring substituents on *N*-arylpiperidines can direct hydroxylation away from the substituted position and improve the product yield [18]. Although we did not observe any aromatic hydroxylation of **3a** during our studies, we observed a significant improvement in regioselectivity with the THPs **3b–d**, in which a substituent was present on the phenyl ring.

Analysis of the products arising from the biotransformation with *p*-methyl **3b** and the *p*-chloro **3c** derivatives revealed that the bacterium was capable of catalysing hydroxylation selectively in the C5-position, providing the C5-*axial* isomers **4b** and **4c** and C5-*equitorial* hydroxylated isomers **5b** and **5c** in significant yields. The *p*-nitro substituted THP **3d** was also regioselectively hydroxylated at C5 and this oxidation was considerably quicker than that of **3b** and **3c**, resulting in an appreciably higher yield of the C5-isomers **4d** and **5d** (Scheme 2). The transformations were found to be highly reproducible with the exception of the *p*-chloro substituted benzyloxypyran **3c**, in which both hydroxylated products were not consistently isolated. In each case, no starting material was recovered, which suggests further breakdown of the THPs by the organism, although this was not investigated further.

**Scheme 3:**
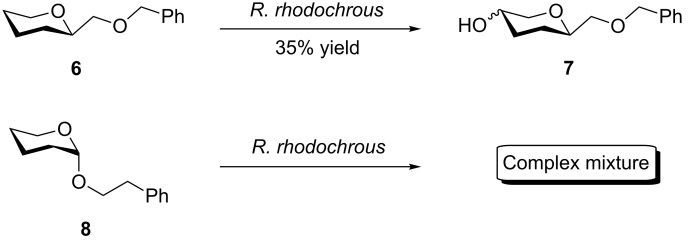
Influence of substrate structure and linker length on the regioselectivity of hydroxylation.

As reported previously with other hydroxylating systems, it is often possible to modify the selectivity of microbial hydroxylation by minor alterations of the substrate structure [16–17]. Although exposure of **3a** to the resting cell suspension resulted in a complex mixture of hydroxylation products, compound **6** demonstrates that insertion of one methylene unit was sufficient to improve the regioselectivity dramatically. THP **7** was isolated in excellent yield (35%) and as the sole regioisomer (Scheme 3).

At this stage it remained unclear as to whether the gain in regioselectivity observed with **6** was due to the increased length of the ether linkage or due to its spatial orientation. NMR evidence confirmed that the ether in **3a–d** sits in the axial position due to the anomeric effect. In the case of substrate **6**, the ether sits in the sterically favoured equatorial position. In order to investigate this effect, substrate **8** (Scheme 3), which combines the axial positioning with the longer ether chain, was synthesised and subjected to the resting cell suspensions. A complex mixture of inseparable products was obtained. This result suggested that the length of the linker was not the most important factor influencing the regioselectivity.

### Transformation of THF ether derivatives

**Scheme 4:**
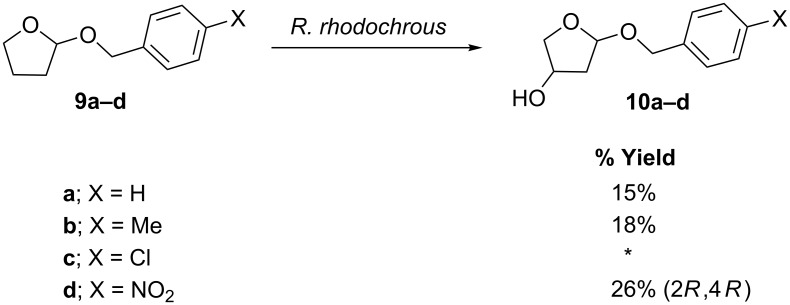
Regioselective hydroxylation following incubation of THF ether derivatives with *Rhodococcus rhodochrous*; *see discussion.

The ability of the organism to catalyse the selective hydroxylation of the THP series **3b–d**, prompted us to investigate the biotransformations of the corresponding racemic tetrahydrofuran **(**THF) compounds **9a–d**. The results, shown in Scheme 4, were largely analogous to those from the THP series. In the case of the THF ring systems, the site of hydroxylation was again determined by NMR, although the relative stereochemistry for all of the derivatives in this series has not yet been determined. In the cases of the substrates **9a** and **9b**, in which the aryl substituents are hydrogen and methyl, respectively, single products were isolated and identified as the C4 hydroxylated products **10a** and **10b**. In the case of the *p*-chloro substituted 2-benzyloxytetrahydrofuran **9c**, 14% of mixed hydroxylated furan derivatives were isolated along with 29% of *p*-chlorobenzoic acid. The isolation of the benzoic acid derivative suggests that benzylic hydroxylation takes place, leading to cleavage of the unstable acetal [19–20]; however, this was not investigated further and the oxidation products were not characterised. The most interesting transformation from this series was that of 2-(*p*-nitrobenzyloxy)tetrahydrofuran (**9d**), which was selectively oxidised to provide product **10d** in substantially higher yield (26%) than the other derivatives in the series.

Following the apparent increase in regioselectivity induced by the insertion of a methylene unit into THP **6**, the organism was challenged with the corresponding THF derivative. In this case, however, the transformation produced only a small amount of a complex mixture of hydroxylated products, which was not purified.

These transformations demonstrate a definite bias towards C5 hydroxylation on the THP series and a preference for C4 hydroxylation on the THF series.

### Enantioselectivity of hydroxylation

The product resulting from the transformation of the *p*-nitro substituted THF derivative **9d** was chosen for some initial screens with the aim of investigating the enantioselectivity of hydroxylation. The MTPA (Mosher) ester **12d** was prepared on reaction of alcohol **10d** with (*R*)-methoxytrifluorophenylacetic acid in the presence of DCC and pyridine. Only one diastereomer was clear in the ^1^H NMR, but examination of the ^19^F NMR clearly showed two fluorine resonances from which an enantiomeric excess of 93% was calculated. The *S*-camphanic ester **11d** was prepared in a 76% yield resulting from the reaction of the alcohol **10d** with *S*-camphanic acid chloride in the presence of pyridine. ^1^H NMR analysis revealed that the resulting ester was a single diastereomer, and the absolute stereochemistry of the THF portion was confirmed by X-ray crystallography and found to be 2*R*,4*R*.

The two products arising from the hydroxylation of THP derivative **3d** showed high optical rotation values, and their enantioselectivity was investigated further. Mosher’s esters **13d** and **14d** of THP alcohols **4d** and **5d**, respectively, were prepared in good yield under the same conditions as those employed for alcohol **10d**. The ^19^F NMR of Mosher’s ester **14d** showed a single peak suggesting an enantiomeric excess of greater than 95%. It should be noted, however, that authentic samples of the enantiomers are not available and it is possible that the resonances may overlap. Examination of the Mosher’s ester **13d** revealed two resonances in the ^19^F NMR with approximately equal peak area. These preliminary results suggest that the organism catalyses the axial hydroxylation of both enantiomers of **3d**, providing **4d** as a mixture of enantiomers.

### Nature of the hydroxylating enzyme

One of the aims of this study was to investigate the nature of the hydroxylating enzyme in an attempt to establish whether the catalytic protein was a cytochrome P450. Inhibition studies with 1-aminobenzotriazole and metyrapone showed that hydroxylation was inhibited by both of these compounds. These cytochrome P450 inhibitors are known to block the activity of the enzyme by different mechanisms [10,12] and are taken to be evidence that a cytochrome P450 system operates in these transformations.

## Conclusion

*Rhodococcus rhodochrous* has been shown to mediate the regio-, diastereo- and enantioselective hydroxylation of unactivated C–H bonds on selected THF and THP derivatives. We have demonstrated that the introduction of substituents onto the aryl ring of the THP ethers can dramatically affect the regioselectivity of hydroxylation. Most notably, exposure of the *p*-nitro substituted THP ether **3d** to resting cells provided the C5-*equatorial* and C5-*axial* regioisomers in 18% and 21%, respectively. Similarly, the organism catalysed the regioselective hydroxylation of *p*-nitro substituted THF ether **10d** providing the C4-hydroxylated product as the sole regioisomer.

Our investigations into the enantioselectivity of hydroxylation revealed that the *p*-nitro THF derivative was hydroxylated selectively, providing the 2*R*,4*R* derivative exclusively. The organism catalysed the enantioselective hydroxylation of *p*-nitro THP **3d**, providing *cis*-hydroxylated **5d** as the sole diastereomer. In contrast, our results indicate that the organism catalyses the axial hydroxylation of both enantiomers of **3d**, providing **4d** as a mixture of enantiomers. As alcohol **4d** showed a strong optical rotation value, this result requires further investigation.

Future efforts will focus on the identification, cloning and heterologous expression of cytochrome P450 genes from this organism to allow for more detailed investigations. It is envisaged that these transformations could be used to produce important hydroxylated, polyfunctionalised chiral intermediates.

## Supporting Information

File 1 Experimental procedures and characterisation data of synthesised, previously unknown compounds.
