# Supplementary material for: Regio- and stereoselective oxidation of unactivated C–H bonds with Rhodococcus rhodochrous
Source: Beilstein J Org Chem. 2012 Apr 3;8:496–500. doi: 10.3762/bjoc.8.56 (PMC3326629; doi:10.3762/bjoc.8.56)
Supplement: File 1 — Experimental procedures and characterisation data of synthesised, previously unknown compounds. [file Beilstein_J_Org_Chem-08-496-s001.pdf]

Supporting Information

for

Regio- and stereoselective oxidation of unactivated C–H bonds with *Rhodococcus rhodochrous*

Elaine O'Reilly[1], Suzanne J. Aitken[1], Gideon Grogan[2], Paul P. Kelly[1], Nicholas J. Turner[1] and Sabine L. Flitsch*[1]

Address: [1]School of Chemistry, Manchester Interdisciplinary Biocentre, The University of Manchester, 131 Princess Street, Manchester M1 7ND, UK, Fax: +44 (0)161 2751311, Tel: +44 (0)161 3065172 and [2]Department of Chemistry, University of York, Heslington, York, YO10 5DD, UK, Tel: +44 (0)1904 328256

Email: Sabine Flitsch* - sabine.flitsch@manchester.ac.uk

* Corresponding author

Experimental procedures and characterisation data of synthesised, previously unknown compounds

General experimental. **S2**

Maintenance and growth of the microorganisms.. **S3**

General biotransformation procedure... **S3**

Compound characterisation... **S4**

trans-2-(4-methylbenzyloxy)-5-hydroxytetrahydropyran (**4b**). S4

cis-2-(4-methylbenzyloxy)-5-hydroxytetrahydropyran (**5b**). S4

trans-2-(4-chlorobenzyloxy)-5-hydroxytetrahydropyran (**4c**). S4

cis-2-(4-chlorobenzyloxy)-5-hydroxytetrahydropyran (**5c**). S4

trans-2-(4-nitrobenzyloxy)-5-hydroxytetrahydropyran (**4d**). S5

cis-2-(4-nitrobenzyloxy)-5-hydroxytetrahydropyran (**5d**). S5

trans-2-(benzyloxymethyl)-5-hydroxytetrahydropyran (**7**)...S5

2-benzyloxy-4-hydroxytetrahydrofuran (**10a**). S5

2-(4-methylbenzyloxy)-4-hydroxytetrahydrofuran (**10b**)..S6

2-(4-nitro-benzyloxy)-4-hydroxytetrahydrofuran (**10d**). S6

Preparation of 2-(4-nitrobenzyloxy)tetrahydrofuran-4-camphanoate (**11d**). S6

2-(4-nitrobenzyloxy)
tetrahydrofuran-4-yl-(trifluoromethylmethoxyphenylacetate) (MTPA ester) (**12d**). S7

trans-2-(4-nitrobenzyloxy)tetrahydropyran-5-yl-(α methoxy-α-(trifluoromethyl-
phenylacetate)) (**13d**)……………………………………………………………………….. S7

cis-2-(4-nitrobenzyloxy)tetrahydropyran-5-yl-(α methoxy-α-(trifluoromethyl-
phenylacetate)) (**14d**) ………………………………………………………………………..S7

General esterification procedure using DCC coupling method (A)…………………………….....S8

General experimental

All chemicals were purchased from Aldrich Chemical Company, Poole, Dorset U.K. unless specified otherwise. ^{1}H and ^{13}C NMR analysis was performed on a Bruker AC250 spectrometer (at 250 MHz and 63 MHz, respectively) and on a WH-360 spectrometer (at 360 MHz and 90 MHz). Spectra were recorded in deuterochloroform unless otherwise specified. Chemical shifts are quoted in parts per million (ppm) and are referenced internally (^{1}H, C*H*Cl_3, 7.27 ppm; ^{13}*C*HCl_3, 77.0 ppm). Coupling constants (*J*) are quoted in hertz. Carbon multiplicity was established by DEPT (distortionless enhancement by polarisation transfer). ^{19}F NMR analysis was performed on a Bruker AC 250

spectrometer. Mass spectra were recorded by using electron impact (EI) on a Finnigan 4600 instrument for nominal-mass and a Kratos MS50TC instrument for high-resolution spectra. Thin-layer chromatography was performed on glass sheets coated with silica gel Merck $60F_{254}$ (0.24 mm, Art. 5715). Components were detected by UV (254 nm) and visualised by treating the plate with ammonium molybdate solution and heating. Flash chromatography was carried out on silica gel (Merck 9385, Kieselgel 60). Optical rotations were recorded in chloroform.

Maintenance and growth of the microorganisms

Rhodococcus rhodochrous NCIMB 9703 was obtained from the National Collection of Industrial and Marine Bacteria, Aberdeen, U.K. The organism was maintained on agar plates at 4 °C and these were subcultured at regular intervals. A loop of bacteria was used to inoculate 50 mL of sterile basal salts medium [S1] with 0.05 g L^{-1} ferrous sulfate heptahydrate and 4 g L^{-1} sodium pyruvate, added through a sterile filter, in a 250 mL Erlenmeyer flask. After three days of agitation on an orbital shaker at 250 rpm at 30 °C, each culture was used to inoculate 500 mL of the same medium with 0.05 g L^{-1} ferrous sulfate heptahydrate and 5 mL octane per 500 mL of culture added, in a 2 L Erlenmeyer flask. This culture was grown for five to seven days. The cells were harvested by centrifugation (10 000 rpm for 30 minutes) and washed with phosphate buffer (50 mM, pH 7.0).

General biotransformation procedure

Resting cell transformations were carried out by resuspending washed cell mass in a one-tenth growth volume of phosphate buffer (50 mM, pH 7.0). A solution of 100 mg substrate in 1 mL ethanol was prepared and this was added to the resting cell culture (200 mL) of *Rhodococcus rhodochrous* NCIMB 9703. After 16 hours incubation as above, the cells were removed by centrifugation and the supernatant was extracted into ethyl acetate. Purification of metabolites was routinely carried out by flash silica chromatography with petroleum ether/ethyl acetate or dichloromethane/ethyl acetate as eluent.

[S1] Solution A: ammonium chloride 2.0 g L^{-1}; potassium dihydrogen phosphate 3.1 g L^{-1}, dipotassium hydrogen phosphate 8.2 g L^{-1}, yeast extract 0.1 g L^{-1}, tryptone 0.1 g L^{-1}; Solution B: magnesium sulfate 0.05 g L^{-1}, manganese sulfate 0.05 g L^{-1}, calcium chloride 0.01 g L^{-1}, ammonium molybdate 0.01 g L^{-1}. Solutions autoclaved separately and mixed when cool.

***trans*-2-(4-Methylbenzyloxy)-5-hydroxytetrahydropyran (4b).** Yield 10 mg, 9%; δ_H (360 MHz, $CDCl_3$) 1.60 (2H, m, C(3)H_A & C(4)H_A), 2.04 (2H, m, C(3)H_B & C(4)H_B), 2.34 (3H, s, Me), 3.47 (1H, dddd, *J* 0.5, 2.0, 3.5, 12.0, C(6)H*eq*), 3.80 (1H, br. s, C(5)H), 4.00 (1H, dd, *J* 2.0, 12.0 C(6)H*ax*), 4.46 (1H, d, *J* 12, C(8)H_A), 4.70 (1H, d, *J* 12, C(8)H_B), 4.78 (1H, dd, *J* 0.5, 2.5, C(2)H), 7.18 (4H, m, aromatic); δ_C (90 MHz, $CDCl_3$) 21.6, 25.6, 26.2, 65.4, 65.6, 76.4, 97.0, 128.4, 129.5, 135.3, 137.8; *m/z* (EI) 222.1256, found 222.1256.

***cis*-2-(4-Methylbenzyloxy)-5-hydroxytetrahydropyran (5b).** Yield 20 mg, 18%; $[\alpha]_D^{20}$ = +7 (*c* 1.0, $CHCl_3$); δ_H (360 MHz, C_6D_6) 1.44 (3H, m, C(5)OH, C(3)H_A, C(4)H_B), 1.70 (2H, m, C(3)H_B, C(4)H_B), 2.11 (3H, s, CH_3), 3.39 (1H, m, C(5)H), 3.48 (1H, ddd, *J* 1.7, 4.3 & 10.7, C(6)H*eq*), 3.54 (1H, dd, *J* 8.8 & 10.7, C(6)H*ax*), 4.37 (1H, d, *J* 12.0, C(8)H_A), 4.59 (1H, dd, *J* 2.6 & 3.0, C(2)H), 4.71 (1H, d, *J* 12.0, C(8)H_B), 7.01 (2H, d, *J* 8, aromatic), 7.25 (2H, d, *J* 8, aromatic); δ_C (90 MHz) 21.6, 28.6, 30.1, 66.0, 66.5, 69.3, 96.7, 128.5, 129.5, 135.2, 137.9; *m/z* (EI) 222.1256, found 222.1263.

***trans*-2-(4-Chlorobenzyloxy)-5-hydroxytetrahydropyran (4c).** Yield 9 mg, 8%; $[\alpha]_D^{20}$ = −11 (*c* 1.6, $CHCl_3$); δ_H (250 MHz) 1.60 (2H, m, C(3)H_A & C(4)H_A), 2.04 (2H, m, C(3)H_B & C(4)H_B), 3.48 (1H, m, C(6)H*eq*), 3.81 (1H, br. m, C(5)H), 3.97 (1H, dd, *J* 2 & 12.0, C(6)H*ax*), 4.47 (1H, d, *J* 12.0, C(8)H_A), 4.70 (1H, d, *J* 12.0, C(8)H_B), 4.77 (1H, dd, *J* 2.5 & 2.5, C(2)H), 7.24 (4H, m, aromatic); δ_C (63 MHz) 25.0, 25.6, 64.8, 65.1, 68.1, 96.6, 128.4, 129.0, 133.3, 136.3; *m/z* (EI) 242.0710, found 242.0707.

***cis*-2-(4-Chlorobenzyloxy)-5-hydroxytetrahydropyran (5c).** Yield 13 mg, 12%; δ_H (360 MHz) 1.83 (5H, m, C(3)H_2, C(4)H_2 & C(5)OH), 3.67 (2H, br. m, C(6)H_2), 3.79 (1H, br. m, C(5)H), 4.50 (1H, d, *J* 12.2, C(8)H_A), 4.72 (1H, m, C(2)H), 4.77 (1H, d, *J* 12.2, C(8)H_B), 7.33 (4H, m, aromatic); δ_C (63 MHz) 27.9, 65.3, 66.0, 68.0, 96.3, 128.4, 129.0, 133.3, 136.2. The compound was insufficiently pure for optical rotation measurement.

***trans*-2-(4-Nitrobenzyloxy)-5-hydroxytetrahydropyran (4d).** Yield 22 mg, 21%; $[\alpha]_D^{20}$ = +36 (*c* 1.2, $CHCl_3$); δ_H (360 MHz) 1.70 (2H, m, C(3)H_A & C(4)H_A), 1.70 (1H, br. s, C(5)OH), 2.13 (2H, m, C(3)H_B & C(4)H_B), 3.53 (1H, m, C(6)H*eq*), 3.99 (1H, dd, *J* 2.2 & 11.8, C(6)H*ax*), 4.65 (1H, d, *J* 13.5, C8-H_A), 4.85 (1H, m, C(2)H), 4.89 (1H, d, *J* 13.5, C(8)H_B), 7.55 (2H, d, *J* 8.8, aromatic), 8.24 (2H, d, *J* 8.8, aromatic); δ_C (90 MHz) 25.5, 26.2, 65.2, 65.7, 68.3, 97.7, 124.1, 128.2, 146.1, 147.8; *m/z* (EI) 253.0950, found 253.0951.

***cis*-2-(4-Nitrobenzyloxy)-5-hydroxytetrahydropyran (5d).** Yield 19 mg, 18 %; $[\alpha]_D^{20}$ = −44 (*c* 0.7, $CHCl_3$); δ_H (360 MHz) 1.88 (5H, m, C(3)H_2, C(4)H_2 & C(5)OH), 3.63 (1H, dd, *J* 8 & 11, C(6)H*ax*), 3.70 (1H, ddd, *J* 1.2, 4 & 11, C(6)H*eq*), 3.80 (1H, m, C(5)H), 4.63 (1H, d, *J* 13.4, C(8)H_A), 4.76 (1H, dd, *J* 3.1 & 3.3 C(2)H), 4.90 (1H, d, *J* 13.4, C(8)H_B), 7.55 (2H, m, aromatic), 8.24 (2H, m, aromatic); δ_C (63 MHz) 27.8, 29.5, 65.2, 66.0, 67.6, 96.8, 123.5, 127.7, 145.5, 147.7; *m/z* (EI) 253.0950, 253.0950.

***trans*-2-(Benzyloxymethyl)-5-hydroxytetrahydropyran (7).** Yield 38 mg, 35%; δ_H (360 MHz) 1.55 (4H, m), 2.12 (5H, m, C(5)OH, C(3)H_2 & C(4)H_2), 3.13 (1H, dd, *J* 10.4 & 10.6, C(6)H*ax*), 3.45 (3H, m, C(7)H_2 & C(2)H), 3.70 (1H, m, C(5)H), 4.03 (1H, ddd, *J* 2.2, 4.8 & 10.6, C(6)H*eq*), 4.53 (1H, d, *J* 12.2, C(8)H_A), 4.58 (1H, d, *J* 12.2, C(8)H_B), 7.29 (5H, m, aromatic); δ_C (90 MHz) 27.0, 32.4, 66.2, 72.6, 72.7, 73.3, 76.4, 127.5, 127.7, 128.8, 138.0; *m/z* (EI) 222.1256, found 222.1257.

2-Benzyloxy-4-hydroxytetrahydrofuran (10a). Yield 16 mg, 15%; δ_H (360 MHz) 1.95 (1H, br. s, C(4)OH), 2.14 (1H, dddd, *J* 1, 2.2, 5.5 & 14.4 C(3)H_A), 2.29 (1H, ddd, *J* 2.6, 6.4 & 14.4, C(3)H_B), 3.87 (1H, d, *J* 9.8, C(5)H), 4.04 (1H, dd, *J* 4.2 & 9.8, C(5)H), 4.53 (1H, d, *J* 11.8, C(7)H_A), 4.59 (1H, br. m, C(4)H), 4.77 (1H, d, *J* 11.8, C(7)H_B), 5.43 (1H, dd, *J* 2.5 & 5.5, C(2)H), 7.34 (5H, m, aromatic); δ_C (90 MHz) 43.3, 69.9, 72.1, 74.3, 103.6, 128.1, 128.3, 128.8, 138.3; *m/z* (EI) 194.0943, found 194.0943.

2-(4-Methylbenzyloxy)-4-hydroxytetrahydrofuran (10b). Yield 19 mg, 18%; δ_H (360 MHz) 2.13 (1H, dddd, *J* 1.5, 2.5, 5.5 & 14.5, C(3)H_A), 2.27 (1H, dddd, *J* 0.5, 2.5, 6 & 14.5, C(3)H_B), 2.37 (3H, s, CH_3), 3.87 (1H, dddd, *J* 0.5, 1.5, 1.5 & 10, C(5)H_A), 4.04 (1H, ddd, *J* 0.5, 4 & 10 C(5)H_B), 4.49 (1H, d, *J* 11.5 C(7)H_A), 4.60 (1H, m, C(4)H), 4.72 (1H, d, *J* 11.5, C(7)H_B), 5.42 (1H, dddd, *J* 0.5, 0.5, 2.5 & 5.5, C(2)H), 7.18 (2H, m, aromatic), 7.25 (2H, m, aromatic); δ_C (93 MHz) 21.0, 42.8, 69.2, 71.7, 73.7, 102.8, 127.9, 128.9, 134.7, 137.3; *m/z* (EI) 208.1099, found 208.1101.

2-(4-Nitrobenzyloxy)-4-hydroxytetrahydrofuran (10d). Yield 28 mg, 26%; $[\alpha]_D^{20}$ = +80 (*c* 1.5, $CHCl_3$); δ_H (250 MHz, $CDCl_3$) 1.97 (1H, br. m, C(4)OH), 2.15 (1H, dddd, *J* 1.2, 2.6, 5.4 & 14.4, C(3)H_A), 2.27 (1H, ddd, *J* 2.6, 6.0 & 14.4, C(3)H_B), 3.83 (1H, ddd, *J* 1.5, 1.5 & 10, C(5)H_A), 3.96 (1H, ddd, *J* 0.5, 4 & 10, C(5)H_B), 4.57 (1H, d, *J* 13, C(7)H_A), 4.58 (1H, m, C(4)H), 4.80 (1H, d, *J* 13, C(7)H_B), 5.38 (1H, dd, *J* 2.5 & 5.5, C(2)H), 7.46 (2H, m, aromatic), 8.17 (2H, m, aromatic); δ_C (63 MHz) 42.7, 68.1, 71.4, 73.9, 103.6, 123.5, 127.7, 145.6, 147.2; *m/z* (EI) 239.0794, found 239.0794.

Preparation of 2-(4-nitrobenzyloxy)tetrahydrofuran-4-camphanoate (11d).

Acid chloride (1.2 mol equiv) and pyridine (2 mol equiv) were added to a stirred solution of the alcohol **10d** (1 mol equiv) in anhydrous dichloromethane under an atmosphere of argon. Dimethylaminopyridine (trace) was added if the reaction was sluggish. On complete consumption of alcohol, the reaction mixture was diluted with dichloromethane and washed with citric acid (5% aqueous solution), saturated sodium hydrogen carbonate solution, brine and water. The organic extracts were dried over anhydrous $MgSO_4$, filtered, concentrated under reduced pressure and purified by flash chromatography. Yield 76%; δ_H (360 MHz) 0.99 (3H, s, Me), 1.08 (3H, s, Me), 1.15 (3H, s, Me), 1.72 (1H, m), 1.95 (1H, m), 2.06 (1H, m), 2.32–2.51 (3H, m, C(3)H_2 & 2 x camphane CH_2), 3.98 (1H, d, *J* 10.8, C(5)H), 4.14 (1H, dd, *J* 4.5 & 10.8, C(5)H), 4.64 (1H, d, *J* 13.2, C(7)H_A), 4.85 (1H, d, *J* 13.2, C(7)H_B), 5.44 (1H, dd, *J* 2.3 & 5.5, C(2)H), 5.53 (1H, m, C(4)H), 7.51 (2H, m, aromatic), 8.25

(2H, m, aromatic); *m/z* (EI) required 419.1580, found 419.1573; 419 (0.8%, M^+), 238 (4, ester cleaved), 137 (59, $O_2NC_6H_4CH_2O$).

2-(4-nitrobenzyloxy)tetrahydrofuran-4-yl-(trifluoromethylmethoxyphenylacetate) (12d). MTPA ester **12d** was prepared following the DCC coupling method A from alcohol **10d** and α-trifluoromethyl-α-methoxyphenylacetic acid. δ_H (250 MHz) 2.23 (1H, dddd, *J* 1, 2.5, 5.5, 15, C(3)H_A), 2.42 (1H, ddd, *J* 2.5, 7.0 & 15, C(3)H), 3.54 (3H, q, J_{HF} 1, OMe), 4.07 (2H, m, C(5)H_2), 4.58 (1H, d, *J* 13, C(7)H_A), 4.80 (1H, d, *J* 13, C(7)H_B), 5.35 (1H, dd, *J* 2.5 & 5.5, C(2)H), 5.58 (1H, m, C(4)H), 7.25–7.52 (7H, m, aromatic), 8.18 (2H, d, *J* 8.8, aromatic); δ_C (90 MHz) 39.8, 55.8, 68.7, 71.3, 76.9, 103.7, 120.0, 124.1, 125.2, 127.6, 128.3, 129.0, 130.2, 132.3, 145.7, 147.8, 166.8; *m/z* (EI) 455.1192, found 455.1192.

***Trans*-2-(4-nitrobenzyloxy)tetrahydropyran-5-yl-(α-methoxy-α-(trifluoromethylphenylacetate)) (13d).** MTPA ester **13d** was prepared following the DCC coupling method A from alcohol 4d and α-trifluoromethyl-α-methoxyphenylacetic acid. δ_H (250 MHz) 1.82 (3H, m & 2.24 (1H, m(C(3)H_2 & C(4)H_2), 3.53 (ddd, J_{HF} 1, OMe), 3.59 (ddd, J_{HF} 1, OMe'), 3.70 (ddd, *J* 2.0, 2.0 &13, C(6) H*eq*), 3.78 (ddd, *J* 2.0, 2.0 &13, C(6) H*eq*'), 3.95 (1H, dd, *J* 2.0 & 13, C(6) H_{ax}), 3.97 (1H, dd, *J* 2.0 & 13, C(6)H_{ax}'), 4.60 (1H, d, *J* 13.5, C(8) H_A), 4.61 (1H, d, *J* 13.5, C(8) H_A'), 4.83 (2H, m, C(8)H_B, & C(8) H_B & C(2)H or C(5)H), 5.28 (1H, br. m, C(2)H or C(5)H), 7.37–7.88 (7H, m, aromatic); δ_C (63 MHz) 21.9 (t, C3 or C4), 22.2 (t, C3' or C4'), 24.6 (t, C3 or C4), 24.8 (t, C3' or C4'), 55.1 (q, OMe'), 55.3 (q, OMe), 61.0 (t, C6 or C8), 61.2 (t, C6' or C8'), 69.9 (d, C2 or C5), 96.2 (d, C2 or C5), 96.3 (d, C2' or C5'), 121.0 (s), 123.6 (d), 125.6 (s), 127.1 (d), 127.4 (d), 127.7 (d), 128.3 (d), 131.8 (s), 132.2 (s'), 145.3 (s), 147.3 (s), 165.9 (s), 166.0 (s'); δ_F (235 MHz) −72.1 & −72.3; *m/z* (EI) required 470.1427, found 470.1427.

***Cis*-2-(4-nitrobenzyloxy)tetrahydropyran-5-yl-(α-methoxy-α-(trifluoromethylphenylacetate)) (14d).** MTPA ester 14d was prepared following the DCC coupling method A from alcohol 5d and α-trifluoromethyl-α-methoxyphenylacetic acid. $[\alpha]_D^{25}$= +8 (*c* 0.65, MeOH); δ_H (250 MHz) 1.93 (4H, m, C(3)H_2 & C(4)H_2), 3.54 (3H, ddd, J_{HF} 1.2, OMe), 3.77 (2H, m, C(6)H_2), 4.58 (d, *J* 13.5, C(8)H_A'),

4.60 (d, *J* 13.5, C(8)H_A'), 4.60 (d, *J* 13.5, C(8)H_A) 4.75 (1H, m, C(2)H or C(5)H), 4.85 (1H, m, C(8)H_B), 5.07 (1H, m, C(2)H or C(5)H), 7.46 (7H, m, aromatic), 8.19 (2H, d, *J* 8.8, aromatic); δ_C (63 MHz) 24.3 (t, C3 or C4), 27.7 (t, C3 or C4), 27.9 (t, C3' or C4'), 55.3 (q, OMe), 61.5 (t, C6' or C8'), 62.3 (t, C6 or C8), 67.6 (t, C6' or C8'), 67.7 (t, C6 or C8), 69.6 (d, C2 or C5), 96.3 (s), 96.9 (d, C2 or C5), 120.8 (s), 123.5 (d, aromatic), 125.6 (s), 127.1 (d, aromatic), 127.6 (d, aromatic), 128.4 (d, aromatic) 129.6 (d, aromatic), 131.9 (s), 145.2 (s), 147,2 (s); δ_F (235 MHz) −72.1; *m*/*z* (EI) required 470.1427, found 470.1427.

General esterification procedure using DCC coupling method (A)

DCC (1 mol equiv) and acid (1.5 mol equiv) were added to a stirred solution of the alcohol (1 mol equiv), pyridine (1.5 mol equiv) and dimethylaminopyridine (catalytic) in anhydrous dichloromethane under an atmosphere of argon. On complete conversion of the alcohol, the reaction mixture was diluted with ethyl acetate and filtered through cotton wool to remove dicyclohexylurea (DCU). The filtrate was evaporated. The dilution, filtration and evaporation cycle was repeated twice to remove traces of DCU. The filtrate was then washed with citric acid (5% aqueous solution), saturated sodium hydrogen carbonate solution, saturated brine and water. The organic extracts were dried over anhydrous MgSO4, filtered and concentrated under reduced pressure. Yields were quantitative and no further purification was carried out.
